# Transmission of Koala Retrovirus from Parent Koalas to a Joey in a Japanese Zoo

**DOI:** 10.1128/JVI.00019-20

**Published:** 2020-05-18

**Authors:** Md Abul Hashem, Fumie Maetani, Mohammad Enamul Hoque Kayesh, Taiki Eiei, Kyoya Mochizuki, Ayaka Ito, Hiroko Sakurai, Takayuki Asai, Kyoko Tsukiyama-Kohara

**Affiliations:** a Transboundary Animal Diseases Centre, Joint Faculty of Veterinary Medicine, Kagoshima University, Kagoshima, Japan; b Laboratory of Animal Hygiene, Joint Faculty of Veterinary Medicine, Kagoshima University, Kagoshima, Japan; c Department of Health, Chattogram City Corporation, Chattogram, Bangladesh; d Hirakawa Zoological Park, Kagoshima, Japan; e Department of Microbiology and Public Health, Patuakhali Science and Technology University, Barishal, Bangladesh; Ulm University Medical Center

**Keywords:** koala, KoRV-A, KoRV-B, KoRV-C, parents, joey, exogenous, endogenous

## Abstract

Koala retrovirus (KoRV) is of an interest to virologists due to its currently active endogenization into the koala (Phascolarctos cinereus) genome. Although KoRV has frequently been isolated in wild and captive koala populations, its pathogenesis and transmission remain to be fully characterized, and most previous research has concentrated on adult koalas rather than on joeys. Here, we characterized KoRV isolates obtained from a deceased male joey and its parents (animals reared in a Japanese zoo) to investigate KoRV transmission mode and pathogenesis. We sequenced the KoRV long terminal repeat (LTR) and envelope genes isolated from the joey and its parents and found KoRV-A and KoRV-C in genomic DNA from both the parents and the joey. Notably, both parents were also positive for KoRV-B, whereas the joey was KoRV-B negative, further confirming that KoRV-B is an exogenous strain. The KoRV LTR sequence of the joey was considerably closer to that of its sire than its dam. For further characterization, total KoRV, KoRV-A, KoRV-B, and KoRV-C proviral loads were quantified in peripheral blood mononuclear cells from the parents and in blood samples from the joey. Total KoRV, KoRV-A, and KoRV-C proviral loads were also quantified for different tissues (bone, liver, kidney, lung, spleen, heart, and muscle) from the joey, revealing differences suggestive of a distinct tissue tropism (highest total KoRV proviral load in the spleen and lowest in bone). The amount of KoRV-C in the parents was less than that in the joey. Our findings contribute to an improved understanding of KoRV pathogenesis and transmission mode and highlight useful areas for future research.

**IMPORTANCE** KoRV is unique among retroviruses in that one strain (KoRV-A) is undergoing endogenization, whereas the other main subtype (KoRV-B) and another subtype (KoRV-C) are reportedly exogenous strains. Its transmission and pathogenesis are of interest in the study of retroviruses and are crucial for any conservation strategy geared toward koala health. This study provides new evidence on the modes of KoRV transmission from parent koalas to their joey. We found vertical transmission of KoRV-A, confirming its endogenization, but with closer conservation between the joey and its sire than its dam (previous reports on joeys are rare but have postulated dam-to-joey vertical transmission). This is also the first report of a KoRV-B-negative joey from KoRV-B-positive parents, contrasting with the few previous reports of 100% transmission of KoRV-B from dams to joeys. Thus, the results in this study give some novel insights for the transmission mode of KoRV.

## Abstract (precis)

KoRV is unique among retroviruses in that one strain (KoRV-A) is undergoing endogenization, whereas the other main subtype (KoRV-B) and another subtype (KoRV-C) are reportedly exogenous strains. Its transmission and pathogenesis are of interest in the study of retroviruses and are crucial for any conservation strategy geared toward koala health. This study provides new evidence on the modes of KoRV transmission from parent koalas to their joey. We found vertical transmission of KoRV-A, confirming its endogenization, but with closer conservation between the joey and its sire than its dam (previous reports on joeys are rare but have postulated dam-to-joey vertical transmission). This is also the first report of a KoRV-B-negative joey from KoRV-B-positive parents, contrasting with the few previous reports of 100% transmission of KoRV-B from dams to joeys. Thus, the results in this study give some novel insights for the transmission mode of KoRV.

## INTRODUCTION

Koala retrovirus (KoRV) is from the genus Gammaretrovirus in the family *Retroviridae*. KoRV is endemic in both wild and captive populations, and it represents a major threat to koala health (1–6). KoRV is of particular interest to virologists because it exists in both endogenous and exogenous forms. Furthermore, compared with other retroviruses that have been endogenized into mammalian genomes for millions of years, the endogenization of KoRV started relatively recently (around 22,000 to 49,000 years ago) and is apparently still progressing, considering that some regional wild populations in Australia reportedly still do not show 100% prevalence (7, 8).

A total of nine KoRV subtypes (KoRV-A to -I) have been identified up to the present (1), among which KoRV-A and KoRV-B are regarded as the major subtypes. KoRV-A is the endogenous subtype; it is found in wild populations, with prevalences ranging from 27% to 100% (8), and it is also endemic in zoo populations. Conversely, KoRV-B is an exogenous strain; it is widespread in both wild and captive populations and is regarded as a major threat to koala health through its pathogenic association with disease progression (1, 4, 5, 9). Furthermore, KoRV-B appears to be undergoing mutation and is spreading rapidly (1).

The mode of transmission has been investigated for both subtypes. As with other endogenous retroviruses, KoRV-A is integrated into the germ line of the host and transmitted vertically to offspring. Reportedly, this vertical transmission apparently occurs between dams and their offspring (1, 10). In contrast, KoRV-B is transmitted horizontally; an annual transmission rate of 3% has been reported for adult-to-adult koala contact per year, as opposed to a 100% transmission rate for dams to joeys (1, 10). However, much work remains to be done to fully characterize KoRV and its transmission. In particular, reports on KoRV status in joeys are scarce.

We previously reported on KoRV infection dynamics (6), and in another study, we found 100% prevalence for KoRV-A and 60% for KoRV-B at three Japanese zoos (11). An interesting finding in the latter study (11) suggested that sire-to-offspring KoRV-B transmission had occurred in one case, which is not consistent with the findings of Quigley et al. (1) and Xu et al. (4); this suggests the need for further research on transmission to offspring and the association between health impact and KoRV subtype and KoRV pathogenesis in joeys.

The koala population at Hirakawa Zoo in Kagoshima, Japan, was among those targeted in our previous KoRV research. Staff at the zoo recently found a 6-month-old joey which had died and been ejected from its dam’s pouch. The KoRV statuses of the deceased joey’s sire and dam (both KoRV-A and KoRV-B positive) were known from our previous studies. Further examination of the parents and the deceased joey represented an opportunity to expand our knowledge on the disease and its transmission between parents and offspring. Accordingly, in this study, we aimed to improve the understanding of KoRV transmission mode and pathogenesis in joeys by investigating the KoRV status of the parents and their joey and by characterizing KoRV in blood and tissues from the joey.

## RESULTS

### Hematological examination of parents.

The hematology results for the parents are shown in Table 1. All blood parameters were within normal ranges, indicating that the parent koalas were generally healthy.

**TABLE 1:** Hematological data from parent koalas

Parameter^*a*^	Data for koala:	
	1 (sire)	2 (dam)
WBC count (×10^2^/μl)	48	113
RBC count (×10^4^/μl)	353	327
HGB (g/dl)	13.9	11.9
PCV (%)	39.4	35.2
MCV(fl)	111.6	107.6
MCH (pg)	39.4	36.4
MCHC (g/dl)	35.3	33.8
PLT count (×10^4^/μl)	2.9	22.2

a WBC, white blood cell; RBC, red blood cell; HGB, hemoglobin; PCV, packed cell volume; MCV, mean corpuscular volume; MCH, mean corpuscular hemoglobin; MCHC, mean corpuscular hemoglobin concentration; PLT, platelet.

### Postmortem examinations of the joey.

**FIG 1:**
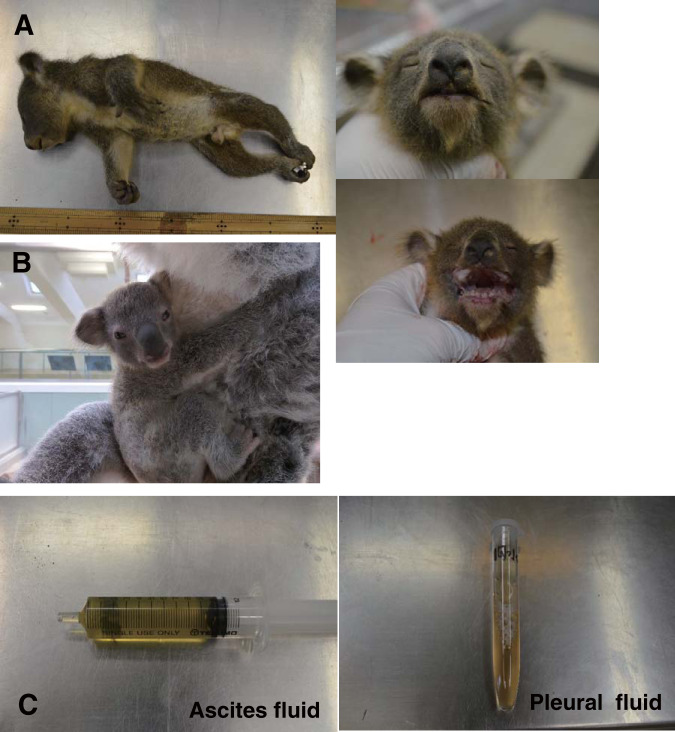
(A) The deceased joey (age, 6 months) and its appearance. (B) A healthy joey (age, 7 months) with its mother. (C) Left, fluid (about 10.2 ml) collected from the peritoneal cavity of the joey at necropsy. Right, pleural fluid collected from the joey’s thoracic cavity at necropsy.

The joey was found dead at 6 months of age, and the appearance of its cadaver is shown in Fig. 1A (height, 29.5 cm; body weight, 483 g). For comparison, a healthy joey (7 months of age) is shown with its mother in Fig. 1B. Necropsy revealed fluid in the thoracic (12.5 ml; relative density, 1.029 kg/m^3^; total protein [TP], 3.4 g/dl; fibrin deposit, +) and peritoneal (10.2 ml; relative density, 1.035 kg/m^3^; TP, 4.2 g/dl; fibrin deposit, +) cavities (Fig. 1C); such findings would not be observed in healthy joeys. Due to the age of the joey and time elapsed since death, the cause of death could not be determined, and tissue specimens of sufficient quality for histopathology could not be obtained.

### Determination of proviral copy numbers in parents and joey.

**FIG 2:**
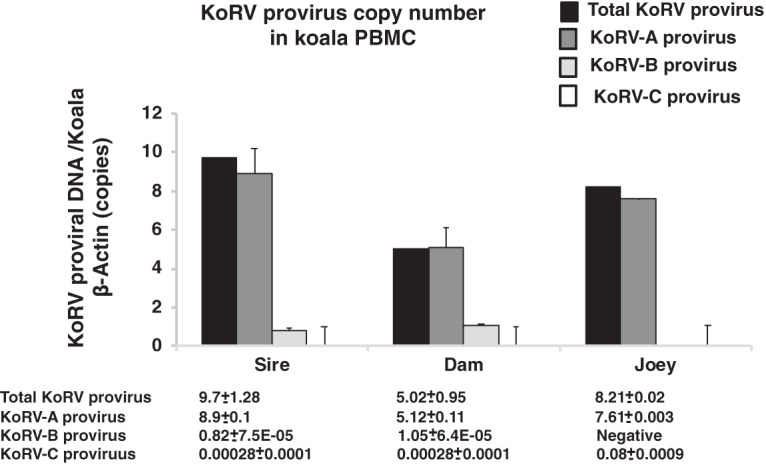
Normalized KoRV proviral load in genomic DNA isolated from koala PBMCs. Normalized total KoRV, KoRV-A, KoRV-B, and KoRV-C proviral loads in parents and total KoRV, KoRV-A, KoRV-B, and KoRV-C proviral loads in samples from the parents (sire and dam) and joey are shown. Provirus copy numbers were quantified by real-time PCR and normalized against corresponding koala β-actin in PBMCs.

Real-time PCR was performed using genomic DNA (gDNA) isolated from peripheral blood mononuclear cells (PBMCs) for the determination of KoRV proviral copy numbers in the parents and joey, with normalization against koala β-actin. Various proviral copy numbers were found in these koalas (Fig. 2). We measured total KoRV proviral load as well as KoRV-A, KoRV-B, and KoRV-C proviral loads. The total proviral and KoRV-A copy numbers were highest in the sire, followed by the joey, and lowest in the dam (Fig. 2). KoRV-B was detected in both the dam and the sire but not in the joey. Furthermore, relatively small amounts of KoRV-C were detected in the joey and its parents, and the amounts in the sire and the dam were less than that in the joey (Fig. 2).

**FIG 3:**
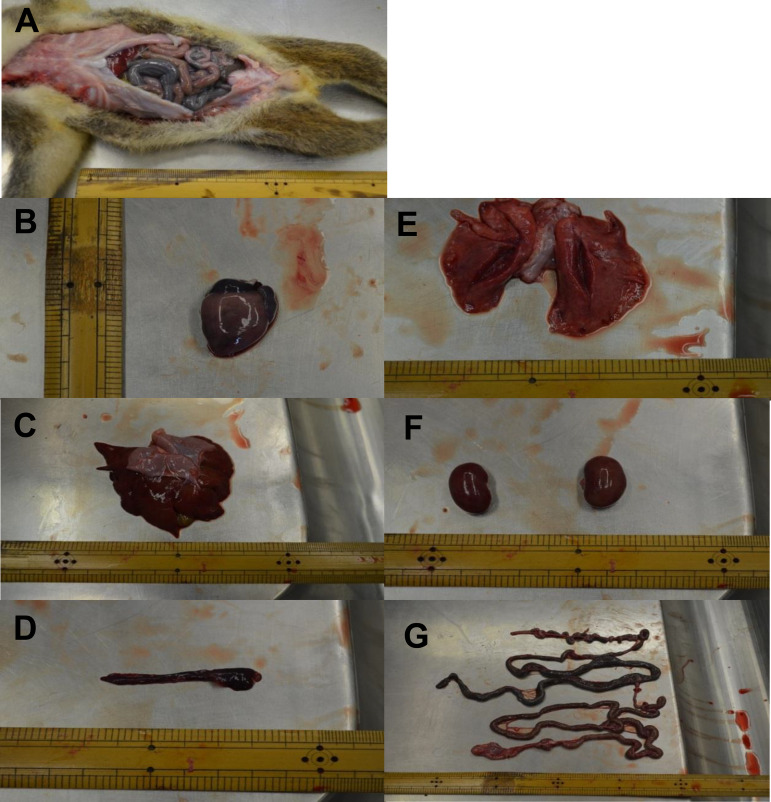
Postmortem examination of the male joey of 6 months old. (A to G) Opening of the abdominal cavity and investigation of the viscera (A), heart (B), liver (C), spleen (D), lung (E), kidney (F), and intestine (G) collected from a baby koala at necropsy.

**FIG 4:**
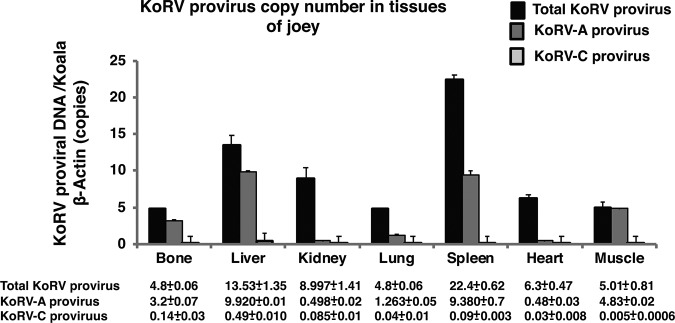
Normalized KoRV proviral load in gDNA isolated from different tissues of a deceased baby koala. Normalized total KoRV, KoRV-A, and KoRV-C proviral loads in different tissues, including bone, liver, kidney, lung, spleen, heart, and muscle are shown. Provirus copy numbers were quantified by real-time PCR and normalized against corresponding koala β-actin in tissues.

To investigate the tissue tropism of KoRV in the joey, the following organs/tissues were collected at necropsy (Fig. 3A): heart (Fig. 3B), lung (Fig. 3C), spleen (Fig. 3D), liver (Fig. 3E), kidney (Fig. 3F), and intestines (Fig. 3G). Using real-time PCR, KoRV proviral copy numbers were determined in gDNA isolated from these samples. The highest total proviral copy numbers were observed in the spleen and the lowest in bone (Fig. 4). Contrastingly, the highest KoRV-A proviral DNA copy number was found in the liver and was the lowest in the heart. KoRV-C proviral load showed a pattern similar to that of KoRV-A, with the highest load in the liver and the lowest load in the muscle (Fig. 4).

### Viral load in plasma.

**FIG 5:**
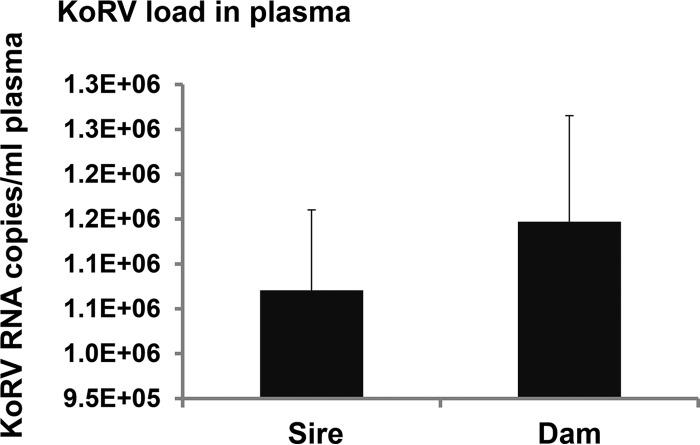
Amount of KoRV RNA copies in the plasma from the sire and dam. KoRV RNA copies per milliliter of plasma were calculated to show the plasma viral load.

Reverse transcription-quantitative PCR (RT-qPCR) was performed to determine viral load in plasma. The viral copy numbers were 1,070,526/ml of plasma in the sire and 1,147,045/ml of plasma in the dam (Fig. 5) and could not be determined in the joey due to postmortem blood coagulation.

### Nucleotide sequence analysis of KoRV in parents and joey.

**FIG 6:**
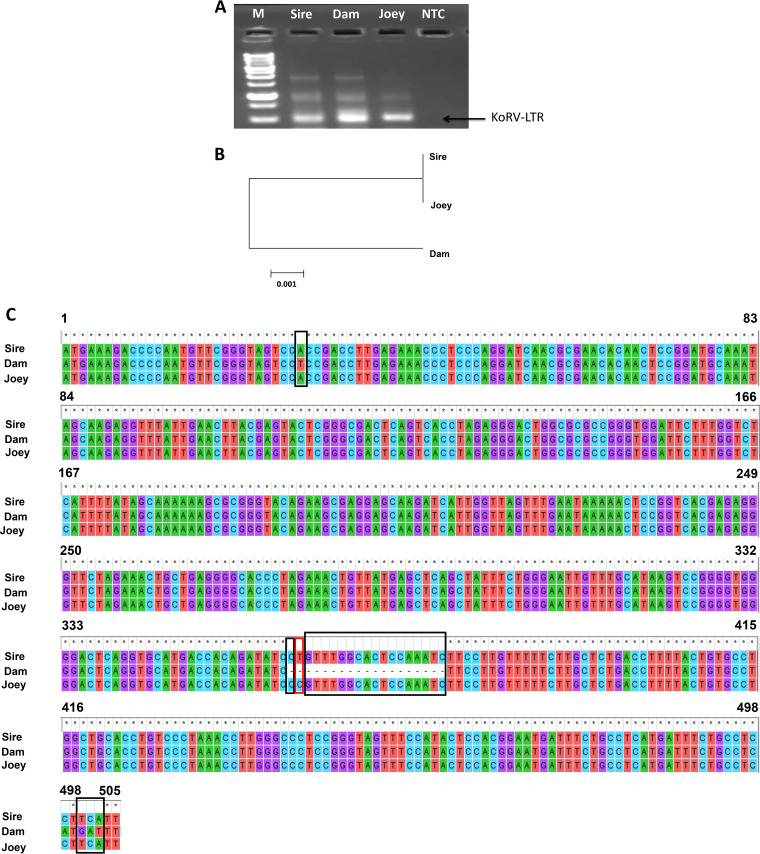
Characterization of long terminal repeats (LTRs) of KoRV in parents and joey. (A) Amplification of LTR gene using genomic DNA from the sire, dam, and joey. Lane NTC, no-template control; lane M, 1-kb marker (GeneDireX, Inc.). (B) A phylogenetic tree was constructed based on the nucleotide sequences of KoRV LTR using the MEGA7 software neighbor-joining method. The scale bar at the bottom indicates the nucleotide distance. (C) LTR multiple-sequence alignment. Black boxes indicate nucleotide differences between the joey with dam only, and the red box indicates nucleotide differences of baby from both the sire and dam. Asterisks indicate conserved sequences.

PCR amplification of sequences with primers U3F and U5R (Table 2) resulted in expected bands of approximately 0.5 kb of the long terminal repeat (LTR) sequence (Fig. 6A). Phylogenetic analysis revealed that the KoRV LTR sequence in the joey (505 nucleotides [nt]) was closer to that of the sire than that of the dam (Fig. 6B). The KoRV LTR nucleotide sequences of the parents and joey were analyzed by multiple-sequence alignment (Fig. 6C). The conserved sequences for the joey and its sire are enclosed in black boxes, and the joey-specific sequences are enclosed within red boxes. These results indicate that the LTR sequence of KoRV in the joey was closer to that of the sire.

**TABLE 2:** Primers used for detection of corresponding KoRV

Target gene (virus)	Primer^*a*^	Primer sequence (5′ to 3′)	Reference(s) or GenBank accession no.
*pol* (KoRV)	Forward	TTGGAGGAGGAATACCGATTACAC	6
	Reverse	GCCAGTCCCATACCT GCCTT	
β-Actin	Forward	AGATCATTGCCCCACCT	6
	Reverse	TGGAAGGCCCAGATTC	
*env* (KoRV-A)	Universal forward	TCCTGG GAACTG GAAAAG AC	11
	Reverse	GGG TTC CCCAAG TGATCT	
*env* (KoRV-B)	Universal forward	TCCTGGGAACTGGAAAAG AC	4, 11
	Reverse	GGCGCAGACTGTTGAGATTC	
	Forward	CGGTGAAGGTTGACGGTATT	
	Reverse	ACCCCAAGGTTCCATAGCTC	
*env* (KoRV-C)	Universal forward	TCCTGGGAACTGGAAAAGAC	KP792564.1
	Reverse	AAGGCTGGTCCCGCGAAGGT	
LTR	U3Forward	AATGAAGGAGGCAGAAATCATGAGGC	NC_039228.1
	Gag 1A reverse	TTCCAGTGATCTAGTGTAAG	
	U5Reverse	ATGAAAGACCCCAATGTTCG	

a The primers for *env* (KoRV-B) were the universal forward and reverse primers or the forward and reverse primers.

**FIG 7:**
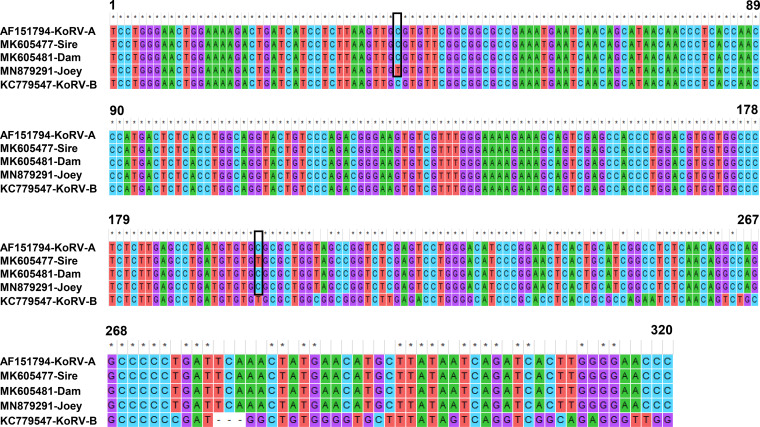
Multiple-sequence alignment of KoRV-A envelope gene. Boxes indicate nucleotide differences between the joey and the sire or dam. Asterisks indicate conserved sequences, and dashes indicate deleted sequences. Published sequences of KoRV-A (GenBank accession no. AF151794) and KoRV-B (GenBank accession no. KC779547) are also shown.

In addition, we sequenced the KoRV-A partial envelope gene (320 nt) from the joey and performed multiple-sequence alignment with the parents’ KoRV-A *env* gene sequences (GenBank accession numbers MK605477 and MK605481) (Fig. 7). Multiple-sequence alignment revealed differences in only two nucleotides, indicated by black boxes (Fig. 7). One point was conserved between both parents and the joey, and one point was conserved between only the dam and the joey. Notably, using two sets of primers (4, 11), we also performed genotype-specific PCR for the detection of KoRV-B in the joey, but no PCR bands of the expected size were observed in any samples from the joey, whereas both the sire and dam were found to be KoRV-B positive (data not shown) with both of the primer sets (4, 11).

## DISCUSSION

In the present study, we investigated the subtype status, tissue tropism, and transmission mode of KoRV in a deceased joey koala from parents previously established to be KoRV-A and KoRV-B positive in our previous studies (6, 11).

For KoRV-A, we speculated that vertical parent-to-offspring transmission had occurred in this case, which is in line with previous findings of 100% vertical KoRV-A transmission in regional koala populations in Australia and the inheritance of KoRV after its endogenization into the genome (1, 11). Notably, the parents were born in captivity, one in a Japanese zoo and one in an Australian zoo. Furthermore, in contrast to reports that offspring inherit KoRV-A from their dams, we found that the joey had a KoRV LTR sequence closer to that of its sire than its dam. Quigley et al. (1) reported on four joeys (age, 0.9 to 2 years) who had all apparently been subject to horizontal infection by their dams. Xu et al. (4) reported KoRV-B statuses for four of five joeys (age range, 1 month to 1 year) in a study population at a Los Angeles zoo and concluded that KoRV-B had been transmitted from the dam to the joey. Differences in points of conservation in multiple-sequence alignment suggest KoRV-A that is continuing to evolve, and these differences are consistent with mutations that can be expected as the endogenized genome is passed from one generation to the next.

Our findings for KoRV-B are of particular interest. The joey was found to KoRV-B negative, although both of its parents were found KoRV-B positive (in both this study and our previous study). Although adult-to-adult transmission of KoRV reportedly occurs at a low rate, the previous (limited) reports on parent-to-offspring transmission suggested that 100% of joeys contract this subtype through horizontal (or *de novo*) infection from their dams. Such transmission could occur through uterine fluids, milk, or pap, which probably originates in the dam’s cecum. KoRV-B-negative joeys with KoRV-B-positive sires have been featured in these reports; however, to our knowledge, this is the first report of a KoRV-B-negative joey from a KoRV-positive dam. As an understanding of transmission is key to any strategy for species conservation, further research on KoRV prevalence and transmission in joeys would appear to be necessary.

The cause of death, and more specifically, whether the cause of death was related to KoRV, could not be established in the limited postmortem examinations that were possible for this joey. The findings of fluids in the thoracic and peritoneal cavities could be consistent with the sort of immunodeficiency or neoplasia reportedly linked with KoRV-B. However, it has to be noted that that the joey in this case was negative for KoRV-B and positive for KoRV-A. According to a previous study, lymphoma and leukemia are found to be the most common forms of neoplasia in both captive and free-living koalas (12). Mortality surveys of wild koalas revealed that these two conditions account for around 3 to 5% of deaths of free-living koalas in the New South Wales and southern Queensland (13–15). Accordingly, the necropsy findings may be consistent with lymphoma or leukemia as the cause of death for the joey in this study. Further research is needed on the possible pathological associations of KoRV-A in joeys.

The joey’s parents were also suggested to be generally in good health, as determined from the hematology examinations in this study and recent veterinary observations, and they had not shown any signs of neoplasia or autoimmune disease, such as the “AIDS-like syndrome” described in KoRV-B-positive koalas, until recently.

We found various proviral loads in the joey organs and tissues examined in this study, indicating that the virus is still active and that replication and *de novo* integration in some organs are ongoing. This indication is consistent with previously reported differential proviral loads in a range of organs for porcine endogenous retroviruses (PERVs) (16, 17, 18). One explanation for such differential proviral loads concerns the status of KoRV-A as a currently endogenizing retrovirus which is still actively infectious, thus causing new insertions in susceptible tissues. Moreover, the larger amount of KoRV-C in the joey than in the parents could reflect progressive integration into the joey’s genome. This result may explain the inconsistency between total KoRV, KoRV-A, and KoRV-C provirus amounts, because they were quantitated using *pol* gene and *env* gene regions. The proviral load in the spleen exceeded the total amounts of KoRV-A and KoRV-C. This result suggests that a strain other than KoRV-A, -B, or -C could be present in the joey’s genome.

Overall, our study confirms that KoRV-B is an exogenous variant of KoRV, and that KoRV-A is inherited by vertical transmission, as has been previously widely reported. However, we suggest that previous assumptions about the 100% dam-to-joey transmission rate for KoRV-B may need to be reexamined. A fuller understanding of transmission and pathogenesis is required to develop any conservation strategies, such as vaccination programs for wild animals, and will be also be of interest to virologists studying the exogenous and endogenous subtypes of this disease. In future research on the koala populations in Japanese zoos, we hope to further expand our knowledge of KoRV and provide answers to some of the questions raised by recent research.

## MATERIALS AND METHODS

### Animals.

The joey was a 6-month-old male found dead and ejected from its dam’s pouch by staff at the Hirakawa Zoo in Japan (Fig. 1A); its cadaver weighed about 450 g. This was the first joey born to this dam. Its sire and dam, identified as KoRV-A and KoRV-B positive in our previous study, were 5 and 3 years old, respectively, at the time of the joey’s death. The dam and sire were born in zoos in Australia and Japan, respectively, and the dam had been reared at the Hirakawa Zoo for 1 year prior to the death of the joey.

### Sample collection.

EDTA-treated whole-blood samples were collected by venipuncture from two the adult koalas (sire and dam) housed at the Hirakawa Zoological Park in Kagoshima, Japan, in accordance with the protocols of the Institutional Animal Care and Use Committee of the Joint Faculty of Veterinary Medicine, Kagoshima University. Tissue samples (bone, liver, kidney, lungs, heart, spleen, and muscle) were collected at necropsy from the dead joey at Hirakawa Zoological Park and stored at −80°C for further characterization. We collected whole-blood samples with heparin or EDTA by venipuncture from 2 koalas (parents) and tissue samples (clotted blood, bone, liver, spleen, lung, kidney, heart, and muscle) from their dead joey at Hirakawa Zoological Park, in accordance with the Institutional Animal Care and Use Committee protocols.

### Hematological examination.

To determine the health status of the parent koalas, a hematological panel was obtained with standard protocols, and this panel included white blood cell (WBC) count, red blood cell (RBC) count, hemoglobin (HGB), packed cell volume (PCV), mean corpuscular volume (MCV), mean corpuscular hemoglobin (MCH), and mean corpuscular hemoglobin concentration (MCHC).

### Preparation of plasma.

Whole-blood samples (EDTA treated) were centrifuged at 3,000 rpm and 20°C for 5 min to obtain plasma samples, which were then stored at −80°C. To measure viral plasma load, viral RNA was isolated from plasma using an Isogen-LS kit (Nippon Gene, Japan), according to the manufacturer’s instructions. To remove any contaminating DNA, extracted RNA was treated with RQ1 RNase-free DNase (Promega), according to the manufacturer’s instructions.

### Isolation of genomic DNA and viral RNA.

Genomic DNA (gDNA) was isolated from EDTA-treated whole-blood samples using a Wizard genomic DNA purification kit (Promega), according to the manufacturer’s instructions. gDNA was isolated from frozen tissue samples using a phenol-chloroform extraction method. The extracted gDNA was used for further characterization.

### PCR, cloning, and sequencing.

PCR was performed using gDNA as a template to amplify the envelope genes of KoRV-A, -B, and -C with genotype-specific primer sets (Table 1), as described previously (4, 9). After the end of the PCR run, the products were further incubated at 72°C for 10 min with LA-*Taq* polymerase. The resulting target PCR fragments were subcloned into the pCR2.1 TOPO vector (Invitrogen) and sequenced.

A nested PCR was performed to determine the nucleotide sequence of the long terminal repeat (LTR) for KoRV-A and the *env* region of KoRV-C, using gDNA as the template. The sequence data of KoRV-C *env* were submitted to GenBank (accession no. MT134110). Tissue was isolated from koala blood or tissue samples using the primer sets in Table 1, as described previously (2, 12). The cyclic conditions for the first PCR were as follows: initial denaturation at 98°C for 2 min, denaturation at 98°C for 30 s, annealing at 57°C for 30 s, extension at 72°C for 1 min, followed by 39 cycles, and final extension at 72°C for 5 min. The second PCR was performed using the product of the first PCR as the template, with annealing at 66°C. The resulting PCR fragments were then subcloned into the pCR2.1 TOPO vector and sequenced.

### Multiple-sequence alignment.

The KoRV-A envelope gene and KoRV LTRs were isolated from the parent koalas and the joey koala and sequenced. The multiple-sequence alignment of the KoRV *env* gene nucleotides and LTR nucleotides was performed using the MEGA7 software (19).

### Phylogenetic analysis.

For phylogenetic analysis of KoRV LTRs, we used KoRV LTR sequences obtained from our study. The phylogenetic tree was constructed on the basis of the neighbor-joining method (20), and evolutionary distances were computed using the *p*-distance method (21). Evolutionary analyses were conducted using MEGA7 software (19).

### Real-time PCR.

Isolated gDNA was used as the template to determine the KoRV proviral DNA copy number by real-time PCR, as described by Kayesh et al. (6), using primer sets (Table 1) with Brilliant-III Ultra-Fast SYBR green qPCR master mix (Agilent Technologies, Santa Clara, CA, USA), according to the manufacturer’s instructions. Amplification and detection were carried out using a CFX Connect real-time PCR detection system (Bio-Rad, USA). The specificity of the PCR was confirmed by melt curve analysis. Standards were generated from prequantified plasmids containing the sequence of the target gene. Koala β-actin was used as an endogenous control for normalization of the KoRV proviral DNA copy numbers. The primer sets used for the detection of koala β-actin are shown in Table 1.

Isolated gDNA was also used for the determination of KoRV-A, KoRV-B, and KoRV-C proviral DNA copy numbers with real-time PCR using genotype-specific primer sets (Table 1) targeting the *env* genes of KoRV-A, KoRV-B, and KoRV-C, using reaction conditions as described previously (11). However, in the case of KoRV-B copy number determination, the reaction conditions were slightly modified, as follows: the initial denaturation at 95°C was for 3 min, and this was followed by 40 cycles at 95°C for 5 s and 55°C for 10 s. PCR specificity was confirmed by melt curve analysis. Standards were prepared from prequantified plasmids containing the target gene sequence. The koala β-actin gene was used as an endogenous control for normalization of the KoRV proviral DNA copy numbers.

### Plasma viral load determination by real-time PCR.

The total KoRV copy numbers in the plasma were determined by real-time PCR, as described previously (6).

### Ethical approval.

This study was performed in accordance with the protocols of the Institutional Animal Care and Use Committee of the Joint Faculty of Veterinary Medicine, Kagoshima University, Japan.

### Data availability.

The sequence data for the KoRV-A envelope gene and KoRV LTRs are available in the GenBank database (accession numbers MN879288 to MN879291).
